# Paths to positive growth in parents bereaved by drug-related death: A mixed-method study

**DOI:** 10.3389/fpsyg.2022.982667

**Published:** 2022-08-25

**Authors:** Kristine Berg Titlestad, Pål Kristensen, Maja O'Connor, Sigurd Hystad, Kari Dyregrov

**Affiliations:** ^1^Department of Welfare and Participation, Faculty of Health and Social Sciences, Western Norway University of Applied Sciences, Bergen, Norway; ^2^Centre for Crisis Psychology, Faculty of Psychology, University of Bergen, Bergen, Norway; ^3^Unit for Bereavement Research, Department of Psychology and Behavioral Science, Aarhus University, Aarhus, Denmark; ^4^Department of Psychosocial Science, Faculty of Psychology, University of Bergen, Bergen, Norway

**Keywords:** drug-related death, parents, bereavement, post-traumatic growth, positive experiences

## Abstract

**Introduction:**

Drug-related deaths (DRDs) are a major public health challenge. Losing a child to a DRD can be a very stressful life event, which places parents at risk of mental and physical health problems. However, traumatic experiences like losing a child to DRD can paradoxically also lead to positive psychological changes. A mixed-method approach was used to understand the complexity of the phenomenon of post-traumatic growth experienced by parents following a DRD.

**Method:**

By combining data from a survey (*n* = 89) and interviews (*n* = 14), we explored positive growth experiences among Norwegian parents. We conducted descriptive analyses of the sample’s demographic characteristics and mean scores for Post-traumatic Growth Inventory (PTGI-SF) items. Hierarchical multiple regression was used to examine the influence of the ability to perform daily activities (WSAS), self-efficacy (GSE-SF), social support (CSS), and symptoms of prolonged grief (PG-13) on the outcome variable of post-traumatic growth (PTGI-SF). Reflexive thematic analysis was applied to analyze the qualitative data. Finally, we integrated the results of the survey and the interviews.

**Results:**

For items measuring post-traumatic growth, parents scored highest on the item “I discovered that I’m stronger than I thought I was” and lowest on the item “I am able to do better things with my life.” Self-efficacy and social support had a statistically significant relation with post-traumatic growth. Two themes were generated from the interviews: (I) new perspectives on life and (II) new paths in life. Even though the “New Possibilities” subscale had the lowest mean score for the PTGI-SF, new paths in life were important for many of the interviewed parents.

**Discussion:**

Parents described traumatic stressors associated with having a child who uses narcotics and hence experienced positive changes even before losing their child. We argue that on an individual level, the consequences of spillover stigma, low self-efficacy, and intrusive rumination can hinder potential post-traumatic growth. On a group level, enhancing network support may increase post-traumatic growth experiences. Hence, parents who have experienced a DRD can benefit from help to activate their social networks and strengthen their self-efficacy.

## Introduction

Traumatic experiences can paradoxically lead to positive psychological changes. Various terms are used for this phenomenon (Calhoun et al., 2010), and we use the term post-traumatic growth (PTG) in this article. PTG is “the experience of positive change that occurs as a result of the struggle with highly challenging life crises” (Tedeschi and Calhoun, 2004b, p. 1). Such changes are not a direct reaction to the traumatic event itself but rather emerge from struggling with the consequences of the event. Hence, PTG results from revaluation processes, when a person reconstructs their beliefs and goals, and how they make sense of their life following a traumatic experience (Tedeschi and Calhoun, 2004a). People who experience traumatic stressors do not necessarily experience growth (Calhoun et al., 2010), but those who do often describe “five factors that define the major domains of PTG: greater appreciation of life and changed sense of priorities; warmer, more intimate relationships with others; a greater sense of personal strength; recognition of new possibilities or paths for one’s life; and spiritual development” (Tedeschi and Calhoun, 2004a).

Considering the Norwegian Directorate of Health (2014, p. 14), drug-related deaths (DRDs) are defined as deaths caused by the intake of substances (intentional and unintentional overdoses), classed as narcotics, and deaths of people linked to drug use in various ways (e.g., violence, suicide, infectious disease, or other health disorders). There is a continuing need to scale up treatment and harm-reduction provisions to prevent DRDs, as the 2022 European Drug Report showed that in 2020 17.4 deaths per million among the adult population in Europe were due to an overdose (European Monitoring Centre for Drugs and Drug Addiction, 2022, p. 16). Sadly, reducing DRDs remains a major public health challenge in all parts of the world. In the US, the number of deaths has reached epidemic proportions reaching a high of over 100,000 deaths in 2021, a yearly increase of 28.5% compared to 2020, during which 78,056 overdose deaths were recorded (National Center for Health Statistics, November 17, 2021). Clearly then, there is a need for attention to drug-related bereavement (i.e., the situation of bereaved left behind after DRDs) as their unique difficulties have been identified in the scientific literature (see later).

Highly stressful events such as bereavement are known to produce high levels of psychological distress (Stroebe et al., 2007; Calhoun et al., 2010; Komischke-Konnerup et al., 2021). Parents who have experienced a drug-related bereavement have a higher mortality rate than non-bereaved parents and parents bereaved by other causes of death (Christiansen et al., 2020). Bottomley et al. (2021) studied the mental health burden associated with overdose-related deaths and found that the overall mental health burden of those bereaved by overdose is substantial compared to those bereaved by sudden natural loss. People bereaved by overdose were almost three times more likely to meet the symptom severity level for prolonged grief disorder (PGD), post-traumatic stress disorder, and major depressive disorder. They also appeared to be at risk of generalized anxiety disorder and suicide (Bottomley et al., 2021). Titlestad and Dyregrov (2022) have also found a strong association between suicidal thoughts and high levels of PGD among bereaved family members, with the highest level of prolonged grief symptoms found among participants in the group who had lost a family member one to 2 years previously. Grief responses that have persisted for more than 6 months are defined as an atypically long period in the ICD-11 (World Health Organization, 2022). After 2 years, high levels of prolonged grief symptoms indicate that those bereaved by DRD have experienced severe grief symptoms lasting much longer than their network and society would expect (Titlestad and Dyregrov, 2022).

Several articles about the parents included in this study have already been published (Titlestad et al., 2020a,b; Titlestad and Dyregrov, 2022), showing that bereaved parents continuously process an overload of stressors (from the time before the loss) as well as the perceived stigma and grief-related emotions and reactions. Parents of a child with a drug dependency described themselves as being in a state of constant preparedness, ready to step in at any time if their child needed help. Their parenting role was extended, and many described themselves as full-time helpers, which was complicated since they did not have an official carer’s license (Titlestad et al., 2020a). Many parents bereaved by a DRD describe the loss of a child as a shock and experience negative changes in the aftermath, such as an overload of grief and emotions, self-stigmatization for failing as a parent, and high levels of prolonged grief symptoms (Titlestad et al., 2020a; Titlestad and Dyregrov, 2022). In sum, the parents described that being bereaved by a DRD was related to significant distress and suffering. Even though losing a child is profoundly disturbing, focusing on the psychological processes that can lead to positive growth is important as this can give service providers an insight into how they can help people cope with major life disruptions (Tedeschi and Calhoun, 2004b).

Tedeschi and Calhoun (2004b) assert that social support may enhance PTG. For those bereaved as a result of an unnatural death, low perceived social support is a major risk factor for mental distress (Scott et al., 2020). Research about the effect of social support on PTG is inconsistent, though some studies show that social support may moderate the relationship between intrusive rumination and PTG (Xu et al., 2019). Dyregrov and Dyregrov (2008, p 123–133) argue that individuals bereaved as a result of an unnatural death and the people in their social networks lack shared understanding of a framework for communication. They explored this phenomenon in light of Berger and Luckmann’s (1991) theories relating to the dynamic influence of individuals and groups on the social context, as well as Briggs’s (1986) communication and interaction model. According to Dyregrov and Dyregrov (2008, p. 125), such theories are essential to understanding the challenges faced by the bereaved and their supporters. Tedeschi and Calhoun (2004b) have described a model illustrating that specific individual characteristics may increase the likelihood of experiencing positive growth. As with Dyregrov and Dyregrov (2008), they highlight characteristics that include openness about the experience, the ability to talk about personal topics, and managing distressing emotions through constructive cognitive processes (Tedeschi and Calhoun, 2004b).

In their “model of growth in grief,” rumination (i.e., repetitive thoughts about the incident) is also underlined as playing an important part for the bereaved (Calhoun et al., 2010). Though rumination is often a burden, it can be helpful for dealing with emotional reactions, distracting the bereaved from the most painful aspects of the loss (Eisma and Stroebe, 2017) and, in the long run, can be part of a growth process (Tedeschi and Calhoun, 2004b). Calhoun et al. (2010) distinguished between intrusive rumination and rumination that is more deliberate and argued that deliberate rumination can predict a greater degree of PTG. In Stroebe and Schut’s (1999) dual process model of coping with bereavement, the authors argue that both types of rumination probably coexist, and the bereaved might oscillate between these two types of rumination.

We have identified only one study investigating PTG in people bereaved by DRD (Sperandio et al., 2021). Sperandio et al. (2021) study included 292 participants from 17 countries, with various relationships to the deceased. Their study, which focused on whether self-compassion (i.e., having an emotionally positive self-attitude) serves as a predictor for PTG, confirmed their initial hypothesis, and they found that hope was a powerful mediator (i.e., with higher scores indicating high levels of PTG). Hence, self-efficacy can be considered a personality resource, affecting how a person copes with loss (Bandura, 1982), and self-efficacy is positively associated with problem-focused coping (Konaszewski et al., 2019). The search for meaning appears to be an important cognitive process on the path to PTG (Tedeschi and Calhoun, 2004b). Meaning-making is the process by which people “make sense of the loss or find some compensatory “benefits” or life lessons in it… [and] commonly integrate the event, adapt, and perhaps even grow through the experience” (Neimeyer, 2019). Feigelman et al. (2018) argue that helping others by facilitating support is an important meaning-making strategy for parents bereaved by a DRD. In Titlestad et al. (2020b), parents reported that being needed by their other children and grandchildren is crucial in the meaning-making process.

PTG is a complex term, and DRD bereavement is an understudied topic (Titlestad et al., 2021). To understand the complexity of the phenomenon of PTG following a DRD, we used various methods. Mixed-method research can be defined as “research studies in which a researcher mixes or combines quantitative and qualitative research ideas, approaches, and techniques in a single research study” (Johnson and Christensen, 2016, p. 468). By combining data from a survey and data from interviews, we sought to elaborate, illustrate, and clarify the quantitative results using the qualitative results. We looked to PTG literature describing the consequences for individuals bereaved by a DRD or other unnatural losses, in order to formulate the hypotheses. We also identified sociodemographic characteristics that are related to higher levels of grief symptoms (e.g., female gender, a low level of education, and unemployment (Heeke et al., 2017)). The following research questions guided our exploration:

*Quantitative research questions:* Do parents bereaved by DRDs report PTG post loss, and if so, which factors can explain high levels of PTG?

We hypothesized that high levels of self-efficacy and support are associated with high levels of PTG and that high scores on the WSAS and high levels of prolonged grief symptoms will be associated with low levels of PTG.

*Qualitative research question:* What positive changes do parents bereaved by DRD describe?

*Mixed-method question:* Can integrating quantitative and qualitative data provide a deeper understanding of PTG experienced by parents bereaved by DRD?

## Materials and methods

### Study design and procedures

The END project was launched in 2017 at the Western Norway University of Applied Sciences. The END project’s primary purpose was to understand better the consequences and care needs of individuals bereaved by a DRD. The project applied a mixed-method approach by collecting quantitative data, using a survey, and collecting qualitative data from semi-structured interviews (ResearchGate, 2022).

The purpose of our study was to explore and investigate PTG *post hoc* from the above data using a mixed methodology. We applied a parallel convergent mixed-method research design, with a deductive approach. Data collection and analysis of data from a survey and interviews took place concurrently, and both components of the research design had the same relevance. The quantitative and qualitative data were analyzed independently. Analysis of the interviews was theory-driven as we were looking for elements of PTG as described in the theories. As described by Johnson and Christensen (2016, p. 595), results from the survey interviews were merged in a joint display at the analysis stage. The reporting standards for mixed methods by Levitt et al. (2018) guided this study.

### Recruitment process and participant selection

From March 2018 until December 2018, we invited family members and friends bereaved as a result of a DRD to participate in the main project. A broad recruitment strategy was launched. All Norwegian municipalities received a recruitment flyer *via* email, and we contacted governmental and non-governmental personnel associated with organizations working with those affected by drug use. We used research networks and clinical practice professionals to contact the bereaved, and also recruited participants through conferences and various media channels. The participants were invited to fill in a questionnaire, either on paper *via* post or digitally *via* email. The participants received an email reminder after 14 days.

Ninety-five parents were enrolled for the survey. The inclusion criteria for this study stipulated that each participant had lost a child due to a DRD at least 3 months before participation. No other restrictions were established concerning the time since death. An additional inclusion criterion for interviews was that participants spoke fluent Norwegian. Participants who missed out more than 25% of the items in the questionnaires were excluded [Post-traumatic Growth Inventory questionnaire (*n* = 5); Prolonged Grief Disorder-13 (*n* = 1)].

Since many more parents (*n* = 75) agreed to be interviewed than could be included, parents were selected according to background variables such as gender, age and place of residency (city/village and northern/central/western/southern/eastern part of Norway), the time since death, and the age and gender of the deceased. We looked to Malterud et al. (2016) for guidance on “information power” to ensure the final sample’s adequacy. Hence, after interviewing seven fathers and six mothers, another mother was invited to participate in case gender became relevant to our discussion, and we concluded that we had reached a satisfactory level of information power. The 14 included parents represented 14 deceased persons (Table 1). One parent represented two deceased, and a divorced couple represented one deceased. One mother withdrew for personal reasons, and one of the recruited participants failed to attend the planned interview.

**Table 1:** Demographic and loss-related variables for the survey and the in-depth interview sample.

Variables		*n*	Survey *n* (%)				*n*	In-Depth interviews *n* (%)			
Men/women sample		89	16 (18)/73 (82)				14	7 (50)/7 (50)			
Men/women deceased		89	68 (76.4)/21 (23.6)				14	10 (71.4)/4 (28.6)			
Level of education		89					14				
Primary school			10 (11.2)								
High school			35 (39.3)					3 (21.4)			
College/university			44 (49.4)					11 (78.6)			
Employment		89					14				
Full-time job			31 (34.8)					7 (50)			
Part-time job			13 (14.6)					1 (7.1)			
On sick leave			3 (3.4)					1 (7.1)			
Retired			20 (22.5)					3 (21.4)			
Studying			1 (1.1)					1 (7.1)			
Other			21 (23.6)					1 (7.1)			
Sick leave before death		88	33 (37.1)				14	4 (28.6)			
Sick leave after death		88	67 (75.3)				14	11 (78.6)			
			*M*	SD	Md	range		*M*	SD	Md	range
Age of participant (years)		87	59.2	7	59	45–80	14	58.3	7.6	58	45–75
Age of deceased (years)		86	26.6	6.3	25	18–45	14	27.4	8.7	24	19–45
PG-13 sum score^*^											
	mothers	73	30.9	8.1	31	15–48	7	30.1	3.5	31	23–34
	fathers	16	29.1	11.5	30	15–49	7	32.1	4.7	31	25–39
Months since loss		88	79.9	78.8	60	3–420	14	37.9	38.5	18	3–126

* The PG-13 total score ranges from 11 to 55, with higher scores indicating more severe grief symptoms. A preliminary cut-off score of 35 or more meets the diagnostic criteria for PGD (Pohlkamp et al., 2018). In the survey sample, 28.7% of the parents’ total score was 35 or higher.

### Data collection and analysis

Table 2 shows an overview of the quantitative and qualitative data collection and how the data were analyzed and integrated at the results stage. The END-project survey consisted of 22 background variables and 87 items from different questionnaires. Our choice of items in this study was made on the basis of literature about PTG after bereavement and studies involving parents bereaved as a result of a DRD (Titlestad et al., 2020a,b, 2021; Titlestad, 2021; Titlestad and Dyregrov, 2022).

**Table 2:** Overview of the methodology.

Type of data	**Research question(s)**	**Sample**	**Data collection**	**Analysis**	**Results**
QUAN	Do parents bereaved by DRDs report PTG post loss, and if so, which factors can explain high levels of PTG?	*n* = 89 73 mothers 16 fathers	**Background variables and five instruments:** The **Post-traumatic Growth Inventory (PTGI-SF**; Cann et al., 2010) is an 8-item questionnaire, based on a 6-point scale, ranging from “not at all” (1) to “extremely” (6), with higher scores indicating high levels of post-traumatic growth (PTG). We assessed four subscales of the PTG: “Relating to Others,” “New Possibilities,” “Personal Strength” and “Appreciation of Life.” Internal consistency with eight items was good, and Cronbach’s alpha (*α*) = 0.865. The **General Self-Efficacy Scale-SF (GSE-SF**; Schwarzer and Jerusalem, 1995) measures self-efficacy. A 5-item short version was used in this study (Tambs and Røysamb, 2014), based on a 4-point scale, ranging from “not at all true” to “exactly true,” with higher scores indicating high levels of self-efficacy; this was applied to investigate whether high levels of self-efficacy are associated with high levels of PTG. Internal consistency was good: *α* = 0.861. The **Crisis Support Scale (CSS**; Elklit et al., 2001) measures social support following a crisis. The first five items measure positive support, scored on a 7-point rating scale, ranging from “never” to “always”; a higher total score indicates more support received (Christiansen et al., 2013). The CSS was applied to investigate whether high levels of support are associated with high levels of PTG. Internal consistency was acceptable: *α* = 0.777. The **Work and Social Adjustment Scale (WSAS**; Marks, 1986; Mundt et al., 2002) measures self-experience of whether the event affects the ability to perform daily activities (e.g., work, housework, leisure activities, and maintaining social relationships). This is a 5-item questionnaire, scored on a 9-point scale, ranging from “no impairment” (0) to “very severe impairment” (8). This was used to investigate whether high scores on the WSAS are associated with low levels of PTG. Internal consistency was good: *α* = 0.886. The **Prolonged Grief Disorder-13** (PG-13; Prigerson and Maciejewski, n.d.) measures symptoms of prolonged grief (PG). A 13-item questionnaire is scored by summing the 11 symptom items (cognitive, behavioral, and emotional), rated on a 5-point Likert scale. Total scores range from 11 to 55, with higher scores indicating more severe grief symptoms. This was applied to investigate whether high levels of PG symptoms constitute a predictor of low levels of PTG. Internal consistency for the 11 items was good: α = 0.892.	Descriptive, correlation and regression analyses were performed using SPSS 26. Continuous variables were described by means (M), standard deviations (SD), medians (Md) and range, whereas frequencies and percentages described categorical variables. The PTGI-SF sum score was chosen as the dependent variable since positive changes and predictors for high levels of change constitute the primary outcome of our analyses. Missing scores were imputed using the individuals’ mean for all items completed (for the PTGI-SF, *n* = 1 (one missing); for the PG-13, *n* = 1 (one missing), and *n* = 1 (two missing)). No replacement was provided for background variables. Hierarchical multiple regression analysis was used to examine the influence of the WSAS, GSE-SF, CSS, and PG-13 on the PTGI-SF outcome variable. The PTGI-SF was regressed on the WSAS, GSE-SF, and CSS in the first step, and the PG-13 was included in the second step. A possible curvilinear effect of prolonged grief was also examined by including a polynomial PG-13 term (PG-132) in the third and final step. The PG-13 was centered around its mean prior to computing the polynomial term.	Presentation of PTGI-SF descriptive analyses, correlations and a hierarchical multiple regression analysis
QUAL	What positive changes do parents bereaved by DRD describe?	*n* = 14 seven mothers and seven fathers	**In-depth interviews:** A semi-structured interview guide, based on questions in the survey, was developed for the interviews. The guide consisted of five topics: (1) the time before the death; (2) the loss; (3) stigma from the environment and self-stigma; (4) help, support, and coping; and (5) post-traumatic growth (PTG). Data from the fifth PTG topic were included in this study. The following topics were explored: • What is the most important thing you have been able to do to live on after the death? • What has promoted or inhibited adjustment to life? • Have you experienced that you have changed as a person after the death – and if so, can you describe how? The interviews were carried out between August 27 and December 4, 2018. To synchronize the interview method and pilot-test the interview guide, the third author conducted a trial interview with a bereaved parent, with the first author present. The interview was discussed with the bereaved individual and the research interviewers. The interview guide was adjusted according to discussions after the trial interview and before other in-depth interviews.	Reflexive thematic analysis, as described by Braun and Clarke (2022), is a 6-phase process: (1) familiarization with the data; (2) coding; (3) generating initial themes; (4) reviewing themes; (5) defining and naming themes; and (6) writing up. Each phase builds on the previous one, and the analysis moves back and forth between the phases. After reading and rereading all the interviews to become immersed and familiar with their content, the entire dataset was coded in Nvivo. We collated data to identify broad patterns of meaning illustrated by themes.	Presentation of codes and themes
MIXED	Can integrating quantitative and qualitative data provide a deeper understanding of PTG experienced by bereaved parents following a DRD?	QUAN: *n* = 89 QUAL: *n* = 14	Data from the survey and the in-depth interviews were collected in parallel.	Quantitative and qualitative data were independently analyzed. The mixed-method analysis was completed at the results stage. We used data from the qualitative interviews to elaborate on the quantitative findings.	Presentation of results from the survey and interviews in a joint display

Overall purpose: To explore positive changes among bereaved parents following a DRD and factors that can explain positive changes.

We included the background variables of gender, age, time since loss, and level of education, as these have been considered to be influential factors in previous studies. The Post-traumatic Growth Inventory (PTGI-SF; Cann et al., 2010) was used to measure the dependent variable since the prevalence of PTG experiences and variables associated with high levels of PTG constitute the primary outcome of our quantitative analyses (see descriptions of this instrument in Table 2). The PTGI-SF scale consists of five subscales and was developed for American samples. Blix et al. (2015) argue that religiosity seems less relevant for a Norwegian sample. Hence, in line with Blix et al. (2015), we excluded the “Spiritual Change” subscale (i.e., item 4: “I have a better understanding of spiritual matters,” and item 8: “I have a stronger religious faith”). The General Self-Efficacy Scale-SF (GSE-SF; Schwarzer and Jerusalem, 1995), Crisis Support Scale (CSS; Elklit et al., 2001), Work and Social Adjustment Scale (WSAS; Marks, 1986; Mundt et al., 2002), and Prolonged Grief Disorder-13 (PG-13; Prigerson and Maciejewski, n.d.) are described in Table 2.

### Mixed analysis matrix

Johnson and Christensen (2016, p. 593) have described several strategies and procedures that may potentially be involved in the analytical process of mixing research. The different approaches involve quantitizing and/or qualitizing data, combining the quantitative and qualitative data to create new datasets, or visualizing the quantitative and qualitative findings separately (Johnson and Christensen, 2016, p. 593). In this study, we used the data display strategy to visualize the quantitative and qualitative findings separately. We then compared and integrated the results from the survey and the interviews in a joint display (see Johnson and Christensen, 2016, p. 593–595). We used data from the qualitative interviews to elaborate on the quantitative findings.

### Ethics statement

The END project was approved in February 2018 by the Norwegian Regional Committees for Medical and Health Research Ethics (reference number 2017/2486/REK vest). All participants signed a written informed consent form, which described the purpose of the study and the planned method and procedure. The well-being of the research participants was prioritized throughout the research process. Participants who agreed to take part in an interview also received written information before the interview, and consent and confidentiality protocol and safeguards were repeated verbally in the interviews. The parents decided where the interviews were to be conducted, e.g., in their homes, where they felt safest and most comfortable. In addition, the participants were made aware of the possibility of contacting the project manager if answering questions about difficult life experiences prompted the need to talk to someone afterward. We ensured the participants’ anonymity and confidentiality at all times, and they had the option to withdraw from the study at any time. We also confirmed that all identifying information concerning survey data, transcripts, and recordings would be anonymized and stored on the research server at the university.

## Results

### Quantitative findings

**Figure 1:**
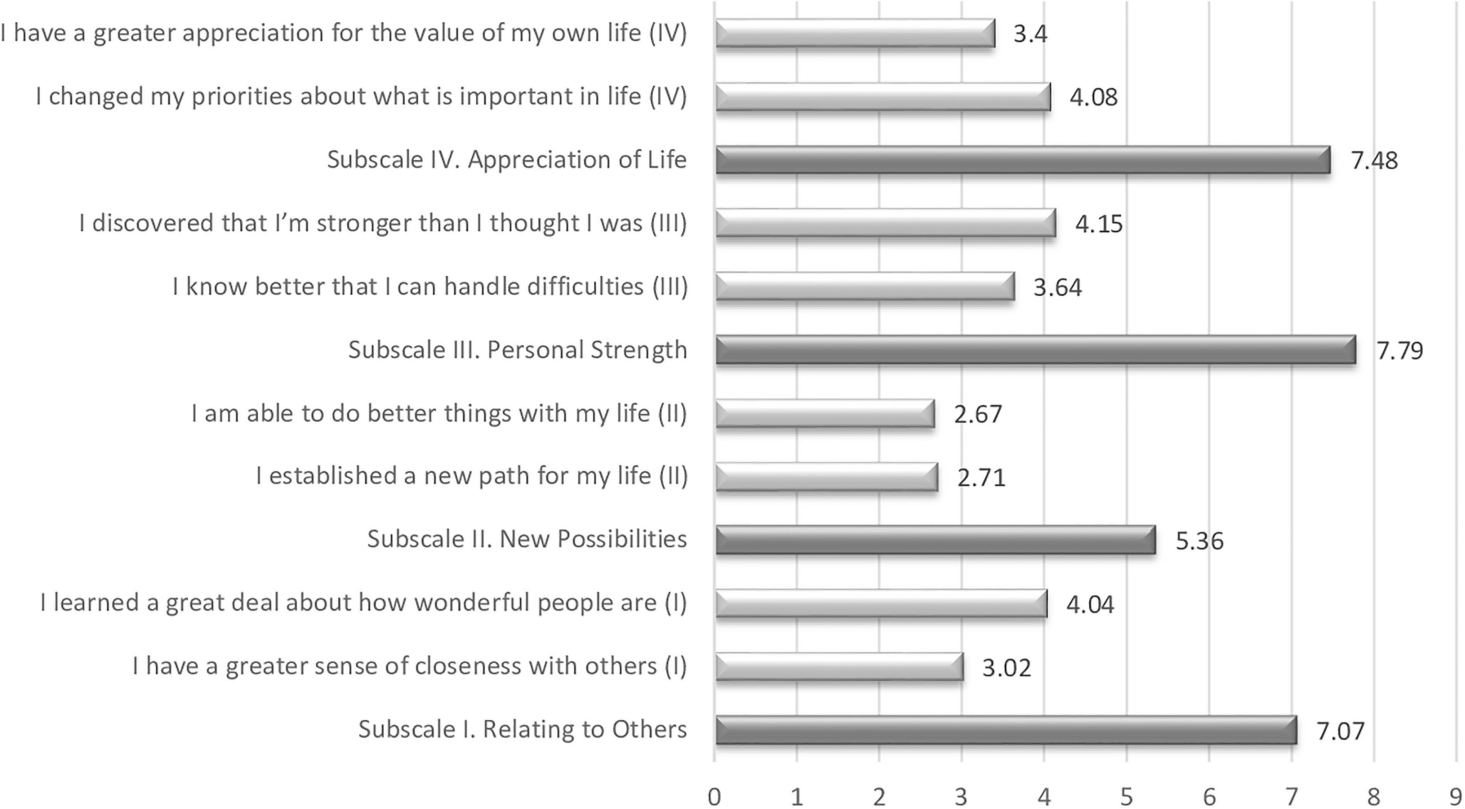
Scores for the four subscales and included items in the PTGI-SF.

The parents’ average PTGI-SF sum score was 27.7 (SD = 9.08), and the scores ranged from 8 to 47. The mean sum score for mothers was 27.7 (SD = 9.2) and was 27.8 (SD = 8.77) for fathers. The mean scores for the eight items and the four included subscales are shown in Figure 1. The participants scored highest on the item “I discovered that I’m stronger than I thought I was” (“Personal Strength” subscale) and lowest on the item “I am able to do better things with my life” (“New Possibilities”) subscale.

Pearson’s product–moment correlations were computed to assess the bivariate relations between the outcome of the PTGI-SF and all potential predictor variables (Table 3). There were no statistically significant correlations between the PTGI-SF and any of the demographic background variables, which were consequently excluded from the regression analysis.

**Table 3:** Mean (SD), internal consistency estimates, and inter-correlations for all study variables (*n* = 89).

Variables		1	2	3	4	5	6	7	8	9
1	Gender	—								
2	Age	0.01	—							
3	Time since death	−0.09	0.25^*^	—						
4	Education	0.06	0.26^*^	−0.10	—					
5	GSE-SF	0.06	0.16	0.08	0.21^*^	*α* = 861				
6	CSS	0.05	0.02	−0.21	−0.03	0.24^*^	*α* = 777			
7	WSAS	−0.31^**^	−0.30^**^	−0.17	−0.09	−0.41^**^	−0.12	*α* = 886		
8	PG-13	−0.08	−0.27^*^	−0.24^*^	−0.24^*^	−0.52^**^	−0.22^*^	0.64^**^	*α* = 892	
9	PTGI-SF	0.01	−0.02	0.14	−0.07	0.40^**^	0.32^**^	−0.25^*^	−0.30^**^	*α* = 865
*M*		18%^a^	59.2	79.9	49%^b^	15.20	24.35	14.45	30.58	27.70
*SD*		—	6.97	78.76	—	2.69	6.65	10.16	8.89	9.08

GSE-SF, The General Self-Efficacy Scale; CSS, The Crisis Support Scale; WSAS, The Work and Social Adjustment Scale; PG-13, Prolonged Grief Disorder; PTGI-SF, Post-traumatic Growth Inventory.

a Indicates percentage of men in the sample.

b Indicates percentage of the sample with a high level of education.

* *p* < 0.05;

** *p* < 0.01.

The WSAS, GSE-SF, and CSS combined explained 21.7% of variations in the PTGI-SF in step 1 [*R*^2^ = 0.217, *F*(3, 85) = 7.867, *p* < 0.001]. As shown in Table 4, both the GSE-SF (*β* = 0.30, *p* = 0.007) and CSS (*β* = 0.24, *p* = 0.018) had a statistically significant relationship with the PTGI-SF. The regression weight of the WSAS was not statistically significant (*p* = 0.33).

**Table 4:** Summary of multiple regression analysis predicting: PTGI-SF (*n* = 89).

			Final model estimates	
Variables	*β*_Step1_	*β*_Step2_	*b*	95% CI
GSE-SF	0.30^*^	0.28^*^	0.95	[0.178, 1.720]
CSS	0.24^*^	0.23^*^	0.32	[0.047, 0.590]
WSAS	−0.10	−0.08	−0.07	[−0.294, 0.157]
PG-13		−0.05	−0.05	[−0.332,0.229]
R^2^	0.217	0.219		
*F*(3, 85)	7.867^**^			
*F*(4, 84)		5.874^**^		

GSE-SF, The General Self-Efficacy Scale; CSS, The Crisis Support Scale; WSAS, The Work and Social Adjustment Scale; PG-13, Prolonged Grief Disorder; PTGI-SF, The Post-traumatic Growth Inventory.

* *p* < 0.05;

** *p* < 0.01.

The PG-13 in step 2 did not explain a statistically significant amount of variation in the PTGI-SF beyond the variables entered in step 1 [Δ*R*^2^ = 0.001, *F*(1, 84) = 0.133, *p* = 0.72]. The GSE-SF (*β* = 0.28, *p* = 0.016) and CSS (*β* = 0.23, *p* = 0.022) remained the only statistically significant predictors in the final model. Both variables explained a comparable amount of variation in the PTGI-SF, with the GSE-SF explaining 5.6% (sr^2^ = 0.056) and the CSS explaining 5.1% (sr^2^ = 0.051).

Adding the polynomial PG-13 term in step 3 did not statistically significantly increase the amount of explained variation in the PTGI-SF [Δ*R*^2^ = 0.003, *F*(1, 83) = 0.267, *p* = 0.61].

### Qualitative findings

Only one father stated that he had not changed after the death. Though the parents were asked about growth after losing a child, some of the changes they described started before death.

Two themes were generated from the data: (I) *new perspectives on life*; and (II) *new paths in life* (Table 5). The themes and the codes are linked and influenced each other (e.g., new perspectives on life and hence new paths in life meant that individuals felt tougher, and it was therefore easier to choose to change).

**Table 5:** Two main themes describing positive changes (*n* = 14).

**Themes**	**Codes**
I. New perspectives on life	(a) Changed attitudes toward what is essential in life
	(b) Increased need for togetherness
II. New paths in life	(a) Felt tougher and braver
	(b) Chose to change
	(c) Increased respect and tolerance for others

The theme of new perspectives on life was related to (a) *changed attitudes towards what is essential in life*; and (b) *increased need for togetherness*. These two codes describe the parents’ new thoughts and points of view.

*Changed attitudes towards what is essential in life* reflect an appreciation of what is meaningful in life. The participants who described changed priorities recognized that there was no need to worry about insignificant matters and expressed that they were less materialistic:

*R: Um, I think the worst has happened, so I’m probably not so, how should I put it, I’m not so concerned that everything must be perfect, no. In my house and at home. (ID 91).*

Being less materialistic also reflected a change in values:

*R: Well, you become a different person in a way, and you have to, you have to learn to live again and think new thoughts and try to find other values in life, right, and some meaning to life, right. So, I’ve probably changed a lot regarding that, and as I said, I do not take everything so seriously anymore. (ID 125).*

New perspectives were also related to strengthening relations with other family members as some of the parents experienced an *increased need for togetherness.* Family members were described as most important, and the participants prioritized and valued them. Some also stated that this unity (the need to take care of the “herd”) was a positive change in the family dynamic which actually started before the child died:

*R: But what has been positive is that we, my daughter and my ex-husband, the father of NN (deceased), have managed to maintain a good relationship and see more of each other now because we have this one grandchild together. At least there is no point in making problems out of it, like getting mad at each other. We just have to try to collaborate and share the experience of being with our grandchild and her mom. (ID 15).*

The theme of new paths in life reflects active sets of changes: (a) *feeling tougher and braver*; (b) *choosing to change*; and (c) *having increased respect and tolerance for others.* These three codes reflect new behavior and actions, changes reported by some of the parents to have occurred before the loss of their child.

Those who described themselves as *tougher and braver* felt that they could handle life’s challenges better. One parent described this as having built a defensive shell to cope with new challenges, so they affected him less. Another explained that being tougher meant being better able to set boundaries for himself, so life did not wear him out:

*R: Well, if I had to rush out in the night and go into some “rat’s nest” and pick him up, I did so… Yes, maybe braver is the word.* (ID 123).

The child’s drug use was all-consuming when the child was alive. Deciding to have things in life other than challenges due to drug use was important for many. For some, *choosing to change* meant that they made a conscious decision to be involved in what they described as meaningful activities (e.g., further education). Others decided to “stop feeling sorry for themselves” and look for positives:

*R: I once stood out on the porch with a coffee before heading to school and thought, because then I was so drained of energy, “[T]his is not working out.” And then there was something in me like, “I want to do this. I will do it. I will get this degree.”* (ID 7).

*Increased respect and tolerance for others* resulted from the fact that they were now less judgmental and more understanding, primarily regarding people who experience drug dependency. Several parents reported that over time they had become humbler and more generous. One parent explained that it was now easier to understand underlying factors and hence had a broader perspective on drug dependency, compared to when their child first started using narcotics:

*R: Yes, I have changed. I might have become a little more humble towards other people and how they live their lives. Now I look a little more behind what is in front of me, look for why things are the way they are, like the negative things… Also, regarding showing consideration or interest for “excluded” people. Previously, I could be a little scared to get too close to them, afraid they were psychotic and dangerous, right? You never know. But I’ve got increased confidence. I’m not so fearful that something will happen to me anymore. Previously, I was much more afraid of other people. I was skeptical of those who were different, and that has changed.* (ID 160).

### Mixed-method findings

Table 6 illustrates integration of the survey and interview data and how the qualitative findings elaborated on the quantitative results. The qualitative finding “felt tougher and braver” expanded on the quantitative result for the highest score on the “Personal Strength” subscale and the strong association between high levels of PTG and self-efficacy. Those who described themselves as tougher and braver reported problem-focused coping when they experienced challenging life experiences. The parents also talked about the personal characteristic “choosing to change,” in the interviews. Taking control of their mindset (like deciding to stop feeling sorry for themselves) and having strategies for coping with their grief as they were focusing on life’s positives also reflect increased personal strength and a high level of self-efficacy.

**Table 6:** Integration of survey and interview data in a joint display.

**Quantitative results (*n* = 89)**	**Qualitative interviews elaborated quantitative findings (*n* = 14)**	**How qualitative findings helped to explain quantitative results**
Highest score on the “Personal Strength” subscale	“Felt tougher and braver”; “Chose to change”	For those interviewed, personal strength involved feeling tougher and braver, and particularly that they felt they could handle life’s challenges better. Personal strength can involve feeling more self-assured (Tedeschi and Calhoun, 1996); the finding “Chose to change” might elaborate on an increase in confidence.
High scores on the “Appreciation of Life” subscale	“Changed attitudes towards what is essential in life”	The qualitative finding reflected an appreciation of what is meaningful in life. Interviewed parents who described changed priorities recognized that there was no need to worry about insignificant matters, and they reported being less materialistic. This reflects an appreciation for smaller aspects of life and taking life easier.
A relatively high score on the “Relating to Others” subscale	“Increased need for togetherness”; “Increased respect and tolerance for others”	A positive change in terms of relating to others was mainly strengthening of existing relationships with family members. Closer relationships with members of participants’ social network were not described. Changes in relating to others also involved greater compassion toward other people who use drugs. Through recognizing and understanding drug dependence as a health issue, many found it easier to relate to people with a drug dependency.
The lowest scores on the “New Possibilities” subscale and the two included items “I established a new path for my life” and “I am able to do better things with my life”	“Chose to change”; “Increased respect and tolerance for others”	Even though the survey responses did not mention establishing a new path in life, the participants interviewed did. New possibilities involved taking a new and different path in life, such as engaging in “meaningful activities,” e.g., further education. The increased respect and tolerance for others that the parents described could involve new relationships.
The strongest association was found between a high score on the PTGI-SF and high levels of self-efficacy.	“Felt tougher and braver”; “Chose to change”	The qualitative findings elaborated that for the interviewed parents, self-efficacy was embedded in personal strength. Self-efficacy is defined as the perception that a person has the capability to react effectively and functionally to environmental demands (Bandura, 1982). Feeling tougher and braver and that they could choose to change constituted a personality resource that could have influenced how individuals coped with their loss.
A strong association was found between a high score on the PTGI-SF and high levels of positive social support.	“Increased need for togetherness”	Through the qualitative findings, we learned that strengthened relationships mainly involved relationships with family members. This indicates that positive social support leading to increased PTG mainly comes from social support provided by family members (although this finding may have important nuances that need to be discussed further).

The quantitative findings showed that the lowest scores on the PTGI-SF were for the “New Possibilities” subscale and the two included items “I established a new path for my life” and “I am able to do better things with my life.” However, in the interviews, the parents reported new paths in life, especially through the possibility of taking a new and different path, such as a career change. In addition, increased respect and tolerance for others characterized the “New Possibilities” subscale, which might lead to new/better relations.

The parents also scored relatively high on the PTGI-SF subscale “Relating to Others,” and the regression analyses showed a strong association between high levels of PTG and positive social support. The qualitative findings highlighted that an “increased need for togetherness” is connected with a greater sense of closeness to other family members, not social networks in general.

## Discussion

In this study, we sought to gain a deeper understanding of PTG experienced by parents bereaved by a DRD, by combining survey results with results from interviews. The highest mean score, indicating high levels of PTG, was found for the “Personal Strength” subscale, and data from the interviews elaborated that these bereaved individuals had increased confidence and felt they could handle life’s challenges better. A low mean score on the “New Possibilities” subscale suggests low levels of PTG. However, data from the interviews were conflicting as recognition of new paths in life was important for many interviewed parents.

We have previously identified high levels of prolonged grief symptoms in the parents included in this study (Titlestad and Dyregrov, 2022). Hence, we highlight again that although PTG was identified, these parents reported that losing a child due to drug use was profoundly disturbing. The correlation analysis showed that high levels of prolonged grief symptoms were associated with low levels of PTG. Though, unlike what others have found when doing more advanced analyses (e.g., Johnsen and Afgun, 2021), we observed no significant association between prolonged grief and PTG when we investigated a possible curvilinear effect of prolonged grief in the hierarchical multiple regression analysis.

Many of the interviewees reported that positive changes started occurring before losing their child, so the major crises for some individuals bereaved by a DRD might be linked to living with and being associated with a child who uses narcotics, and not only the death itself. Since our findings also showed that high levels of self-efficacy are associated with high levels of PTG, we will discuss how self-confidence can be affected by circumstances linked to a person’s drug use. Finally, as high levels of received positive social support are associated with high levels of PTG, we will discuss the potential for shared understanding of a framework for communicating about DRD among parents and people in their network.

### Traumatic experiences linked to the child’s drug use

Our findings shed light on traumatic experiences as a continuous struggle with the consequences of having a child who experiences drug dependency. Having a family member with a dependency can be a long-lasting strain (Lindeman et al., 2021). The meta-ethnography by Lindeman et al. (2021) summarized studies exploring how substance use influences family life. It showed that families constantly have to adapt to their family member’s needs and that new strategies bring hope at first, which soon turns to despair when it becomes clear that these strategies are insufficient. The bereaved parents in our study described having to cope with a child suffering from drug dependency as an enduring overload and being in a state of constant emotional and physical preparedness (Titlestad et al., 2020a). Maltman et al.’s (2019) study of parental grief showed that this burden increases when parents felt they lacked the skills to manage their child’s drug use. In the study presented here, parents reported an increase in personal strength. Through the interviews, we learned that they could handle life’s challenges to a greater extent through feeling tougher and setting boundaries so that life’s challenges did not wear them out. Since the child’s drug use was all-consuming, parents decided to engage in meaningful activities. Changes in “Relating to Others” (e.g., greater compassion toward people who use drugs and strengthened ties with family members) and appreciating smaller aspects of life indicated changed values. Thus, some of the parents in our study described having reconstructed their beliefs and goals, making an effort to engage in meaningful activities before the child died, and experiencing these changes as helpful when it came to coping with the subsequent loss and life in general.

After years of fearing death, many interviewees still reported that death came as a shock (Titlestad et al., 2020a), although a cross-sectional study by Feigelman et al. (2022), comparing individuals bereaved by a DRD (*n* = 115), suicide (*n* = 185,) or sudden natural death (*n* = 103), showed that those bereaved by a DRD anticipated their loved one’s death to a greater extent, while those bereaved by suicide were more shocked. Fearing the death of your child from an overdose, combined with an overload of stress, can be considered as a highly challenging life crisis and hence a traumatic experience (see Tedeschi and Calhoun, 2004b). Feigelman et al. (2022) study supports our findings, indicating that such traumatic experiences are not only linked to the death of a child but also to the consequences of having a child who uses narcotics.

### Consequences of spillover stigma

Having a close relationship with a person who uses drugs and then suffering a drug-related bereavement are linked to stigmatization and self-stigmatization (Dyregrov and Selseng, 2021; Titlestad et al., 2021). Goffman (1963) writes about associative stigma as a spillover; the tainting of an individual in such a way that social discredit affects them to the same degree. Such discrediting can lead to the stigmatized person feeling shame. Self-stigma or internalization of public perceptions can lead individuals to believe that they are incompetent (Sheehan and Corrigan, 2020). Corrigan et al. (2009) have reported that internalized stigma is linked to depression, low self-esteem, and reduced self-efficacy. The parents interviewed in this study have previously reported low self-efficacy due to years of labeling, discrediting, and devaluation as a parent by others and themselves (Titlestad et al., 2020a). The attitudes, norms, and values that people, helpers, bureaucrats, and politicians have toward people who use drugs were internalized in the bereaved, which complicated their grieving process.

We found an important association between belief in one’s own ability to cope with challenges (i.e., self-efficacy) and high levels of PTG. In line with Konaszewski et al. (2019), the results of this study suggest that those who experience PTG have strategies for coping with their grief and believe that they can influence the grieving process by taking control of their mindset, i.e., “choosing to change.” Since self-efficacy can affect how a person copes with a loss (Bandura, 1982), it is essential to help build a sense of self-efficacy among parents bereaved by DRDs, after years of devaluation by themselves and others.

Previously, we have documented that the parents included in this study described shame and self-imposed guilt for failing as a parent, thus triggering rumination (Titlestad et al., 2020a). Service providers need to be aware that deliberate reflection may be helpful as this can help the bereaved make sense of traumatic events and reconstruct a new understanding of others and the world, leading to PTG (Calhoun et al., 2010). Calhoun et al. (2010) argue that failing to rebuild a functional assumptive world belief can be associated with continued high levels of intrusive rumination. Hence, understanding this type of cognitive processing and when it occurs may be crucial to understanding the cognitive routes to PTG (see Tedeschi and Calhoun, 2004b). For the bereaved to cope with loss and be able to adjust to life by oscillating between loss-orientation and restoration-orientation stressors (see Stroebe and Schut, 1999), it is therefore vital to understand that the intrusive rumination they experience may be due to spillover stigma.

### The challenges of providing and receiving support

A strong association was found between high levels of PTG and high levels of positive social support. The qualitative findings elaborated on the quantitative results and showed that the parents had an “increased need for togetherness.” We also learned that strengthened relations were mainly linked to family members. We know from Titlestad et al. (2020b) that the same parents as in this study reported that families with good dynamics before the loss shared their grief and were brought closer together, while many divorced parents who were in conflict before the death experienced a more complicated relationship. As social support is documented as being one of the most important factors for coping with the loss of a loved one (Dyregrov and Dyregrov, 2008), there is a need to shed light on the potential for support for those bereaved by DRD from people other than a family member, i.e., support from social networks.

Through a common understanding that communicating details relating to DRDs is challenging and perhaps extreme for the parties involved, both the bereaved individuals themselves and those who support them can enhance their interaction and communication (see Dyregrov and Dyregrov, 2008). Parents bereaved by a DRD can influence and improve their social relationships by openly communicating their personal needs and educating others on how they can best be supported. Even more importantly, family and friend networks need to be informed of their potential role in healing as respectful and empathetic listeners. To improve such interaction, the bereaved need to know the advantages of openness and how to educate people in their network on the best way to support them. As social support may play a strong role in the development of PTG (see Tedeschi and Calhoun, 2004b), enhancing the relationship between parents bereaved by DRDs and their potential supporters can improve the chances of PTG. Service providers should be aware that bereaved individuals with a high degree of self-efficacy will probably find it easier to stimulate their network by being transparent and open, compared to parents with low self-esteem (see Dyregrov and Dyregrov, 2008).

### Strengths and limitations

The PTGI-SF was one of several other questionaries included in the END survey. Baker et al. (2008) have discussed the challenges associated with instruments that only allow respondents to describe experiences related to positive personal changes. Negative experiences have been reported in several studies (e.g., Titlestad et al., 2020a, 2022). Thus, we argue that one of the strengths of the survey in the END project is that it allows participants to report negative as well as positive experiences.

Cann et al. (2010) have described limitations and areas for caution that should be considered when applying the short form of the PTGI. They argue that the PTGI-SF should be used when a single total score for growth is desired (since two items for each factor might be unreliable in smaller samples) and that several non-English studies have failed to replicate the factor structure in translated versions of the PTGI. In Norway, Blix et al. (2013) used the PTGI-SF in a longitudinal study after the 2011 Oslo bombing. Their results showed that items within the “Spiritual Change” domain came lowest on the PTG factor, resulting in a poor overall model fit. Religiosity seemed less relevant for this Norwegian sample than for the American samples that the scale was developed for. Hence, the two questions in the PTGI-SF on spiritual change did not affect the main findings but improved the overall model fit. We relied on the results of Blix et al. (2013) and assessed PTG with 8 items rather than 10. A consequence of this is that the total score for our participants is not comparable with participants in studies from other countries. Thus, in order to be transparent about the results for the PTGI-SF, we chose to describe scores for factors and single items (Figure 1), as we believe that these results are more informative for readers.

The cross-sectional design used in this study has its limits. It may be challenging to determine whether exposure or the outcome comes first, so we documented associations and not the causality of our findings. The prevalence is derived from a convenience sample, depending on the recruitment method, thus limiting the findings’ generalizability. As no data from the registry of bereaved parents were available, we sought to recruit widely by all possible means for 1 year, resulting in the world’s largest sample of parents bereaved as a result of a DRD.

## Conclusion and implications for practice

This study identified PTG in parents bereaved by DRD and factors that can lead to positive growth. The findings add perspectives to other results from the same sample (Titlestad et al., 2020a, 2022) as they show that intense emotional pain and significant psychosocial impairment can exist alongside positive experiences. We have discussed how having a child who uses narcotics can be a traumatic experience that leads to PTG before the child dies. Thus, the major life crises that led to positive psychological changes for the parents were a continuation of incidents that happened before the loss, followed by the loss of a child due to an unnatural death.

We argue that on an individual level, the consequences of spillover stigma, low self-efficacy, and intrusive rumination may reduce PTG, while on a group level, enhancing network support may be related to PTG experiences. Proactive bereaved individuals can help those in their social networks take the first challenging step in talking about DRDs. Social networks can benefit from being informed that support must be provided on the terms of the bereaved and that listening with respect and empathy can enhance communication with the bereaved (Dyregrov and Dyregrov, 2008). Overall, this study may give service providers greater insight into how to activate parents’ social networks and help them understand how enhancing self-efficacy may increase PTG in the midst of crisis and pain. More research is needed to identify what promotes or inhibits self-efficacy in parents bereaved by DRD and to identify contextual, conditional triggers.

## Data availability statement

The datasets presented in this article are not readily available because the research ethics committee does not approve of sharing the datasets presented in this article. Requests to access the datasets should be directed to kbti@hvl.no.

## Ethics statement

The studies involving human participants were reviewed and approved by The Norwegian Regional Committees for Medical and Health Research Ethics. The participants provided their written informed consent to participate in this study.

## Author contributions

KT, PK, MO'C, SH, and KD conceived this study. Together with other colleagues in the END project. KT and KD collected the quantitative and qualitative data. KT and SH analyzed the quantitative data. KT and KD analyzed the qualitative data. All authors contributed to the article and approved the submitted version.

## Funding

This work was supported by the Research Council of Norway [grant no. 300732] and the Western Norway University of Applied Sciences.

## Conflict of interest

The authors declare that the research was conducted in the absence of any commercial or financial relationships that could be construed as a potential conflict of interest.

## Publisher’s note

All claims expressed in this article are solely those of the authors and do not necessarily represent those of their affiliated organizations, or those of the publisher, the editors and the reviewers. Any product that may be evaluated in this article, or claim that may be made by its manufacturer, is not guaranteed or endorsed by the publisher.
